# Cerebrospinal fluid immune cells appear similar across neuropathic and non-neuropathic pain conditions

**DOI:** 10.12688/wellcomeopenres.20153.1

**Published:** 2023-10-25

**Authors:** Zoe Hore, Jonathan Royds, Ramla Abuukar Abdullahi, Jon Lampa, Adnan Al-Kaisy, Franziska Denk

**Affiliations:** 1 Wolfson Centre for Age-Related Diseases, King's College London, London, England, UK; 2 Guy’s and St Thomas’ Chronic Pain Department, St Thomas Hospital, London, UK; 3 Rheumatology Unit, Department of Medicine, Karolinska Institutet, Stockholm, Sweden

**Keywords:** Human Cerebrospinal Fluid, Neuropathic Pain, CITE-seq, FACS, Postherpetic Neuralgia, Complex Regional Pain Syndrome, Microglia

## Abstract

**Background:**

Microglia have been implicated in the pathophysiology of neuropathic pain. Here, we sought to investigate whether cerebrospinal fluid (CSF) might be used as a proxy-measure of microglial activation in human participants.

**Methods:**

We preformed fluorescence-activated cell sorting (FACS) of CSF immune cell populations derived from individuals who experienced pain with neuropathic features. We sorted CD4+, CD8+ T cells and monocytes and analyzed their transcriptome using RNA sequencing. We also performed Cellular Indexing of Transcriptomes and Epitopes (CITE) sequencing to characterize the expression of all CSF immune cells in a patient with postherpetic neuralgia and in a patient with neuropathic pain after failed back surgery.

**Results:**

Immune cell numbers and phenotypes were not obviously different between individuals regardless of the etiology of their pain. This was true when examining our own dataset, as well as when comparing it to previously published single-cell RNA sequencing data of human CSF. In all instances, CSF monocytes showed expression of myeloid cell markers commonly associated with microglia ( *P2RY12*, *TMEM119* and *OLFML3*), which will make it difficult to ascertain the origin of CSF proteins: do they derive directly from circulating CSF monocytes or could some originate in spinal cord microglia in the parenchyma?

**Conclusions:**

We conclude that it will not be straightforward to use CSF as a biomarker for microglial function in humans.

## Introduction

Neuropathic pain is a type of pain that arises when our nerves are directly affected by damage or disease ( Finnerup *et al.*, 2016). It is particularly resistant to currently available analgesics ( Finnerup *et al.*, 2015) and causes a range of unpleasant sensory symptoms, including burning, and pins and needles. Unfortunately, neuropathic pain is common, highlighted by a recent epidemiological study based on large numbers of participants within UK Biobank, where it was found to affect 9.2% of middle-aged adults ( Baskozos *et al.*, 2023).

Mechanistically, pre-clinical studies have indicated that spinal cord immune cells, so-called microglia, play a role in the emergence of neuropathic pain. The clearest indication of this comes from traumatic nerve injury models in rodents, where clear proliferation of microglia can be observed in the areas where the roots of a damaged peripheral nerve enter the cord. This is a very reliable finding, first observed in the 1970s ( Coyle, 1998), and since repeated many times across many research groups ( Hore & Denk, 2019). There is also a great number of articles which describe that inhibiting this microglial proliferation and associated activation, either pharmacologically or genetically, can reduce neuropathic pain-like behavior in rodents, e.g. ( Fernandez-Zafra *et al.*, 2019; Guan *et al.*, 2016; Ledeboer *et al.*, 2005). Moreover, a recent report even claimed that there might be spinal microglial subpopulations with divergent functions, with one of them necessary for neuropathic pain remission rather than maintenance ( Kohno *et al.*, 2022). 

One issue with the current literature is that, for obvious reasons, its results almost exclusively derive from animal models. There have been efforts to image microglia in humans, using positron emission tomography (PET) imaging of the translocator protein TSPO ( Loggia *et al.*, 2015). However, TSPO is quite widely expressed in other cell types in blood and brain, complicating its use ( Turkheimer *et al.*, 2015) and limiting its utility to specific experimental designs. Accordingly, the search for improved PET tracers for microglia has been gaining significant traction in recent years, with several promising targets such as the P2X7 receptor ( Narayanaswami *et al.*, 2018).

In the meantime, another possibility might be to use cerebrospinal fluid (CSF) as a biomarker for microglial function. CSF is produced by the choroid plexus and fully envelopes our brain and spinal cord in liquid, ensuring that a 1.2kg human brain effectively only “weighs” 45g ( Spector *et al.*, 2015). It supplies the brain with nutrients (e.g. vitamin C), hormones (e.g. leptin) and growth factors (e.g. bdnf) and removes waste products, including those arising from dopamine and serotonin metabolism, from the central nervous system parenchyma ( Spector *et al.*, 2015). CSF also contains and acts as a drainage point for a number of immune cells (e.g. T cells, B cells and monocytes) which carry out surveillance in perivascular, leptomeningeal and ventricular spaces, right next to, but separated from central nervous system (CNS) tissue by various physical barriers, like the glia limitans, pia mater and ependymal cells ( Engelhardt *et al.*, 2017; Louveau *et al.*, 2017). Several groups have previously studied the CSF proteome in pain patients ( Baraniuk *et al.*, 2005; Khoonsari *et al.*, 2018a; Khoonsari *et al.*, 2018b; Lim *et al.*, 2017; Moore & McCrory, 2017; Yuan *et al.*, 2002), including those experiencing neuropathic pain ( Backryd *et al.*, 2018; Lind *et al.*, 2016; Lu *et al.*, 2012). The results have been interesting and point to dysregulation of factors, like chemokines and complements ( Abu Hamdeh *et al.*, 2020; Backryd *et al.*, 2017; Khoonsari *et al.*, 2018b), that are known to be upregulated in rodent microglia in models of neuropathic pain ( Denk *et al.*, 2014; Tay *et al.*, 2017). What is unknown however, is whether these factors are indeed released from CNS microglia and then leak into the CSF or whether they stem from other immune cells, such as monocytes, circulating within the CSF. This is certainly a possibility, with recent single cell RNA sequencing (scRNA-seq) studies of human immune cells ( Farhadian *et al.*, 2018; Heming *et al.*, 2021) indicating that some myeloid cells within the CSF can produce transcripts classically thought of as microglial, e.g. Prdm12 and Tmem119.

Even beyond microglia however, there are indications that it might be informative to examine CSF immune cell profiles in neuropathic pain conditions. Specifically, Natural Killer (NK) T cell numbers have been shown to be differentially upregulated in chronic inflammatory demyelinating polyneuropathy compared to Guillain-Barré syndrome, another inflammatory neuropathy ( Heming *et al.*, 2019). Meanwhile, NK cells were upregulated in Guillain-Barré syndrome compared to the non-neuropathic condition of idiopathic intracranial hypertension (IIH). Conversely, in individuals living with non-inflammatory polyneuropathy, it has been reported that high NK cell numbers might be linked to improved outcomes, since they correlated with reduced mechanical pain thresholds ( Lassen *et al.*, 2021). 

Here, we examined the CSF from further conditions that caused symptoms suggestive of neuropathic pain in the affected individuals: complex regional pain syndrome (CRPS), postherpetic neuralgia (PHN) and failed back surgery syndrome (FBSS). We performed Cellular Indexing of Transcriptomes and Epitopes (CITE)-sequencing, as well as fluorescence-activated cell sorting (FACS) followed by bulk RNA sequencing of sorted immune cell populations. We set out to examine whether 1) the transcriptome of CSF monocytes once again resembled that of microglia and 2) whether there were any stark differences in immune cell populations between these conditions as well as a non-neuropathic condition like IIH.

## Methods

### Participants

Seven individuals gave written informed consent to participate in this study which was approved by HRA and Health and Care Research Wales (REC reference: 19/LO/0037). Neuropathic features were assessed via clinical examination and the help of the DN4 questionnaire. Gender, age and condition of participants are provided in Table 1. CSF was donated prior to implantation of spinal cord stimulation (SCS) neuromodulation devices.

**Table 1.** Characteristics of individuals enrolled in this study.

Gender	Age	Indication
**Male**	53	FBSS
**Female**	73	Postherpetic Neuralgia
**Male**	35	CRPS
**Female**	31	FBSS
**Female**	44	CRPS
**Male**	58	FBSS
**Female**	57	FBSS

### CSF FACS

**Figure 1.**
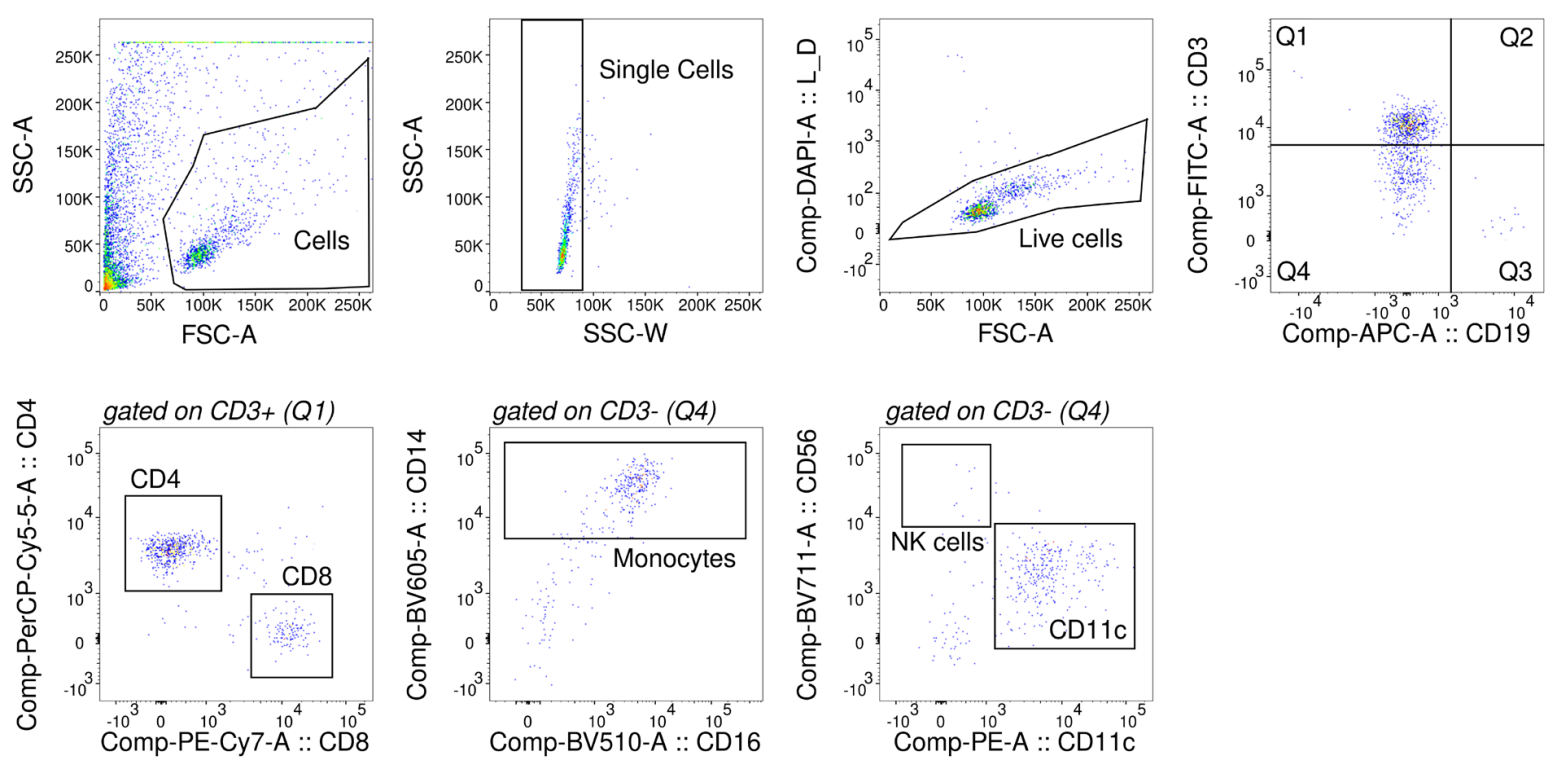
Representative gating strategy for human CSF. FACS was completed within hours of CSF sample collection. Cells were spun down, and labelled with antibodies against T cells (CD3, CD4, CD8), B cells (CD19), monocytes (CD14, CD16, CD11C) and NK cells (CD56). Monocytes and NK cells were gated on CD3, CD19 negative populations. Beads were used for compensation.

5ml of CSF was withdrawn using fluoroscopy guided lumbar puncture. Blood contamination was avoided by discarding the first few drops of sample. The sample was then picked up and transported on ice into the lab within 15-20 minutes. There, it was spun at 200xg for 10 minutes at 4C, after which the supernatant containing CSF proteins was pipetted off. For bulk sequencing, the cells were resuspended on ice in 300µl of filter-sterilized FACS buffer: 2mM EDTA (Thermo Fisher, 15575020), 15mM HEPES (Thermo Fisher, 15630080), 0.4% BSA (Merck, A9418) in HBSS without Ca, Mg or phenol red (Thermo Fisher, 14175095). 2x 30µl were removed for unstained and live/dead single staining controls, while the remaining cells were incubated for 15 minutes on ice with the antibody panel provided in Table 2. After incubation, DAPI was added as a live-dead stain, and cells were sorted on a BD FACS ARIA in the BRC Flow Core at King’s College London. BD Compensation Beads (BD Biosciences, anti-mouse, 552843) were used as single staining controls. The gating strategy is displayed in Figure 1. Three populations (CD4+ T cells, CD8+ T cells and CD16/CD14++ monocytes) were captured in SMARTer cell lysis buffer. The lysis buffer was prepared as described ( Picelli *et al.*, 2014) with minor modifications; first 1µl of RNAse inhibitor (Takara Bio 2313A) and 0.4µl DTT (0.1M from the Thermo Fisher Superscript III kit, 18080051) were added to 18.6µl of 0.2% Triton X-100 (diluted in nuclease-free water from 100% Triton X-100, Thermo Fisher T9284-100ml); then, per sample, 2µl of this mix was supplemented in a DNA lo-bind tube with 1µl 10mM dNTP mix (Fisher Scientific 10319879) and 1µl 10 oligo-dT(30)VN (ordered from Thermo Fisher at 50nM, PAGE purified, sequence: AG CAG TGG TAT CAA CGC AGA GTA CTT TTT TTT TTT TTT TTT TTT TTT TTT TTT TTA G). We prepared several tubes of lysis buffer for each cell population and sorted 50 cells per tube to allow freezing of independent batches at -80C until further processing. Sample processing time was kept to a minimum, with a median time from CSF withdrawal to freezing FACS’d samples on dry ice of 2 hours (min: 1h 45min, max: 3 h). This is key to ensure maximal recovery of monocyte populations ( de Graaf *et al.*, 2011).

**Table 2.** Antibody panel used for CSF FACS. All antibodies, as well as the Fc block, were purchased from BD Biosciences. DAPI was purchased from Merck.

*Laser*	*Cell type*	*Stain or Antibody*	*Catalogue* * number*	*Concentration*
**UV**	Live Dead	DAPI	D9542	3ng/µl
**Violet**	NK cells	BV711 – CD56	563169	1:300
**Violet**	Monocytes	BV605 – CD14	564055	1:200
**Violet**	Monocytes	BV510 – CD16	563829	1:1200
**Blue**	T cells	FITC – CD3	561806	1:50
**Blue**	CD4+ T cells	PerCP Cy5.5– CD4	566321	1:100
**Yellow**	CD8+ T cells	PECy7 – CD8	560917	1:600
**Yellow**	Monocytes/ Dendritic Cells	PE – CD11c	560999	1:300
**Red**	B cells	APC – CD19	561742	1:100
**N/A**	N/A	Fc Block	564220	1:150

For CITE sequencing, we used the Human TBNK TotalSeq-A cocktail from BioLegend (399907). After the first spin of the sample, all the liquid bar the last 100µl was removed. Of that, 40µl of cells were resuspended in the kit’s Cell Staining Buffer for unstained and single staining controls, while the remaining 60µl were incubated with 5µl FcX buffer for 10min at 4C. Subsequently, cells were incubated with the kit’s TBNK cocktail at 1:100 dilution for 30min at 4C. DAPI was then added before sorting for live cells (DAPI+ single events) on a BD FACS ARIA in the BRC Flow Core at King’s College London. Cells were collected in 20µl of filter-sterilized 2% BSA (Merck, A9418) in D-PBS without Ca and Mg (Thermo Fisher, 14190144). Single cell portioning and barcoding was performed by the BRC Genomics Core Facility at King’s College London on a 10X Chromium Platform following manufacturer’s protocols. The two different samples (FBSS and PHN) were processed at different times, which means that direct comparison was constrained by batch effects, only some of which can be overcome computationally.

### Bulk RNA sequencing

Lysed cells were amplified into double stranded cDNA using the SMARTer protocol as described elsewhere ( Picelli *et al.*, 2014). All three cell types (CD4+ T cells, CD8+ T cells and monocytes) derived from one individual were amplified at once. Due to fast degradation of the very low number of cells at -80C, it was impossible to batch control amplifications across patient samples for the cDNA amplification stage. Since library preparation has been found to be a significant source of technical variation ( SEQC Consortium, 2014), batch control was reinstated for subsequent steps, with all samples processed at once within the same library preparation and multiplexed into the same Illumina sequencing lane (150bp, paired-end reads). This was performed by the company Genewiz. 

Reads were pseudo-aligned with kallisto version 0.48.0 to the human genome: Homo Sapiens GCRh38, kallisto index version 10 ( Bray *et al.*, 2016). An average of 29M reads were sequenced per sample, of which an average of 20M reads were successfully aligned. However, given that we only sorted very few cells (i.e. 50) per sample, this amounted to only 5M unique reads on average. See Data_File_1 for alignment statistics for each sample. We considered genes to be detectable in our dataset if their Transcripts Per Million (TPM) value was equal to or more than 1 in all samples of a given cell type. Differential expression was performed by running DESeq2 ( Love *et al.*, 2014) or limma ( Ritchie *et al.*, 2015) in R.

### CITE sequencing

Sequencing was performed on an Illumina NextSeq 500 by the BRC Genomics Core at King’s College London. Both mRNA and reads from antibody-derived tags (ADT) were aligned using 10X Genomics CellRanger. The remaining analyses were performed in R using Seurat ( Hao *et al.*, 2021). For scripts, see Supplementary_Notebook. Briefly, integration of the two CITE-seq datasets was performed using Seurat integration algorithms. We also integrated our data with a previously published scRNA-seq dataset containing CSF samples from patients with IIH ( Heming *et al.*, 2021). To compare annotations between the two datasets, a Sankey plot was generated using the SingleR ( Aran *et al.*, 2019) and SingleCellExperiment packages ( Amezquita *et al.*, 2020).

## Results

CSF was obtained from 7 different individuals living with pain that had neuropathic features. Four had an underlying diagnosis of failed back surgery syndrome (FBSS), two of complex regional pain syndrome (CRPS) and one of postherpetic neuralgia (PHN). In all cases, the pain was intractable enough to make the individuals eligible for a trial of spinal cord stimulation on the National Health Service in the UK.

We quantified immune cell numbers using FACS. The gating strategy is displayed in Figure 1. Live single cells were first gated on CD3 vs. CD19 to distinguish T cells, B cells and non-lymphocytes. CD3+ cells were then divided further into CD4+ and CD8+ T cells. CD3 and CD19 negative cells were gated further on CD14+ and CD16+ double positivity (to identify monocytes), on CD56 (for NK cells) and on CD11C (often used as a dendritic cell marker, but which is likely to label mostly monocytes in the case of CSF). Performing FACS on CSF immune cells is technically very challenging ( de Graaf *et al.*, 2011). Not only does sorting have to proceed very quickly to avoid monocyte loss, but the low number of immune cells in each sample mean that gates have to be placed without the help of fluorescence minus one controls. We therefore focused largely on markers that had very clear positive and negative populations. The exception to this was CD56, which often had very few cells, making gates difficult to place, and CD11C, where a negative population was not always apparent. Nevertheless, in keeping with data from subsequent sequencing, we estimate that between 95-99% of CD14+/CD16+ monocytes were CD11C positive.

**Figure 2.**
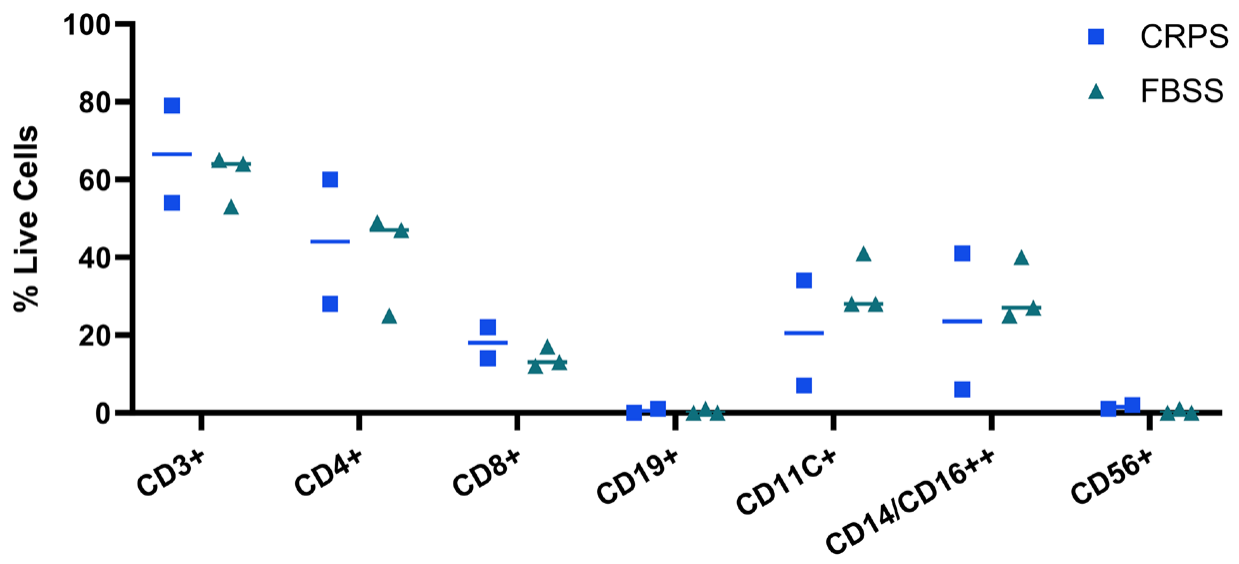
Immune cell populations identified in CSF do not markedly differ in number between CRPS and FBSS. Plotted here is the percentage of total live singlets within each immune cell population identified via FACS using the gating strategy shown in Figure 1: CD3+ T cells; CD3+/CD4+ T cells; CD3+/CD8+ T cells; CD3-/CD19+ B cells; CD3-/CD19-/CD11C+ monocytes; CD3-/CD19-/CD14+/CD16+ monocytes; CD3-/CD19-/CD56+ NK cells. Each dot is a sample derived from a different patient – those with neuropathic pain due to CRPS in blue and due to FBSS in green (n = 5). Lines represent the median.

As already reported by many other research groups before us ( de Graaf *et al.*, 2011), we found that the vast majority of live CSF immune cells consisted of CD3+ T cells (median: 66.5%, min: 54%, max: 79%) and monocytes (median: 23.5%, min: 6%, max: 41%). Most of the T cells were CD4+ (median: 44%, min: 28%, max: 60%), though there was also a fair proportion of CD8+ T cells (median: 18%, min: 14%, max: 22%). No clear differences emerged in live immune cell types across FBSS or CRPS patients ( Figure 2), but of course, this comparison would have been constrained by our small sample size (n = 5) to detect anything but the most unusually large effect sizes.

**Figure 3.**
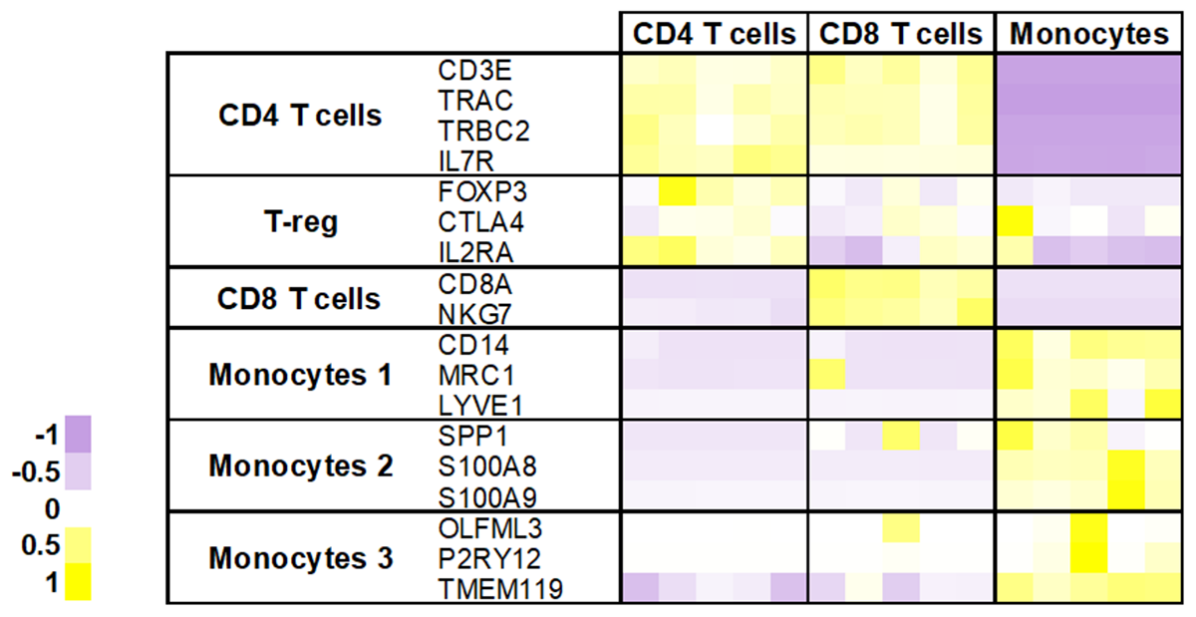
Expression of marker genes for known CSF immune cell populations. Bulk RNA-seq was performed on sorted CD4+ T cells, CD8+ T cells, and monocytes. Plotted here are z-scores of TPM values obtained for a list of known CSF immune cell marker genes selected from ( Heming *et al.*, 2021). Each square represents the expression value of a sample, with three cell populations each derived from n = 5 individuals.

**Figure 4.**
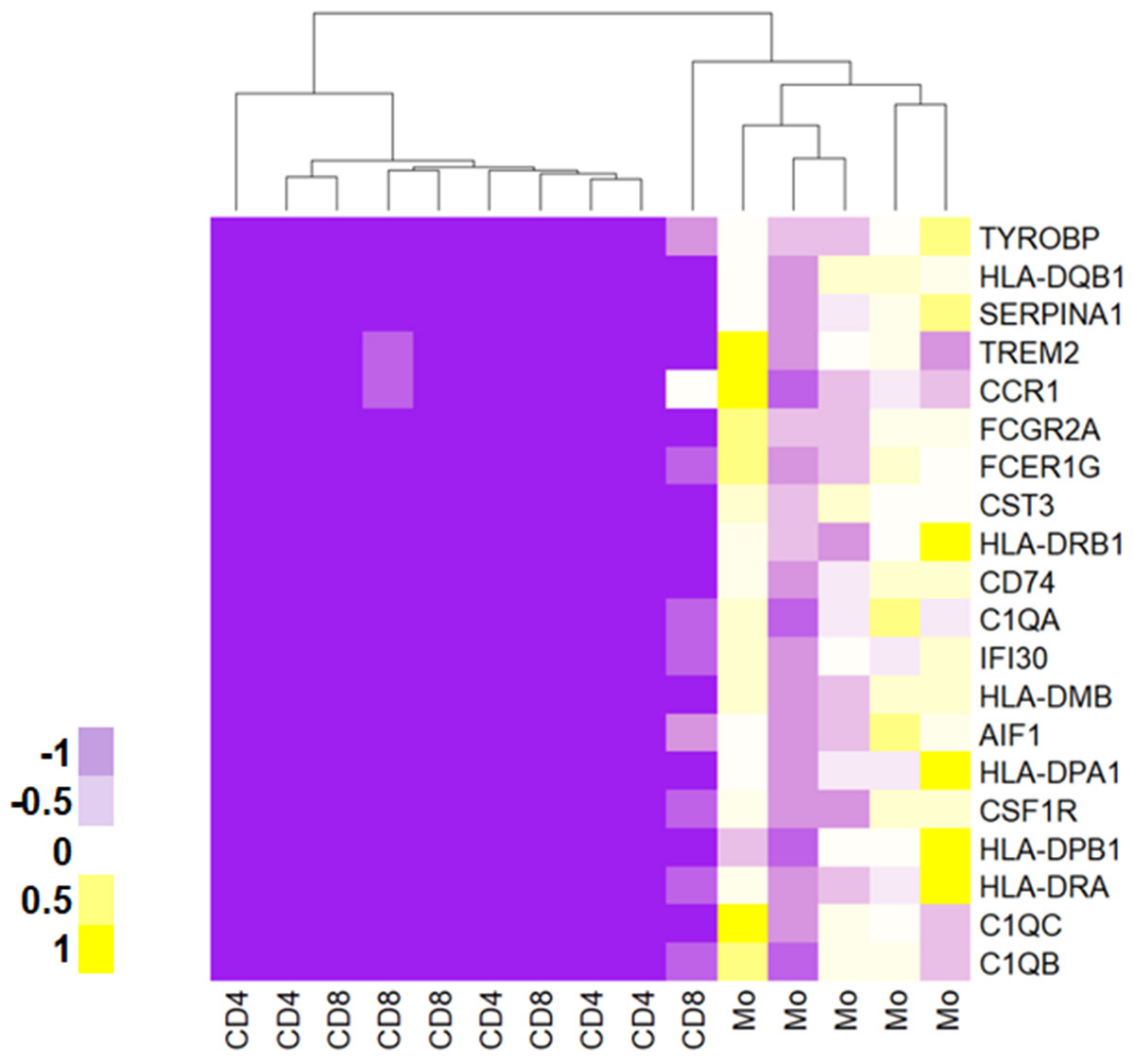
Top 20 most differentially regulated genes using the limma algorithm. As expected, monocyte samples were the most distinct within the bulk RNA-seq data we generated. Plotted here are the 20 most differentially regulated genes, calculated using limma. Each column is a sample. Colors represent z-scores of TPM values. See Data_File_2 for raw values. Columns were re-ordered in R using unsupervised clustering with the hclust function (method = ward.D2). Clustering was performed on all genes which were expressed according to our cut-off (see Methods) and differentially regulated at adj. p < 0.05.

**Figure 5.**
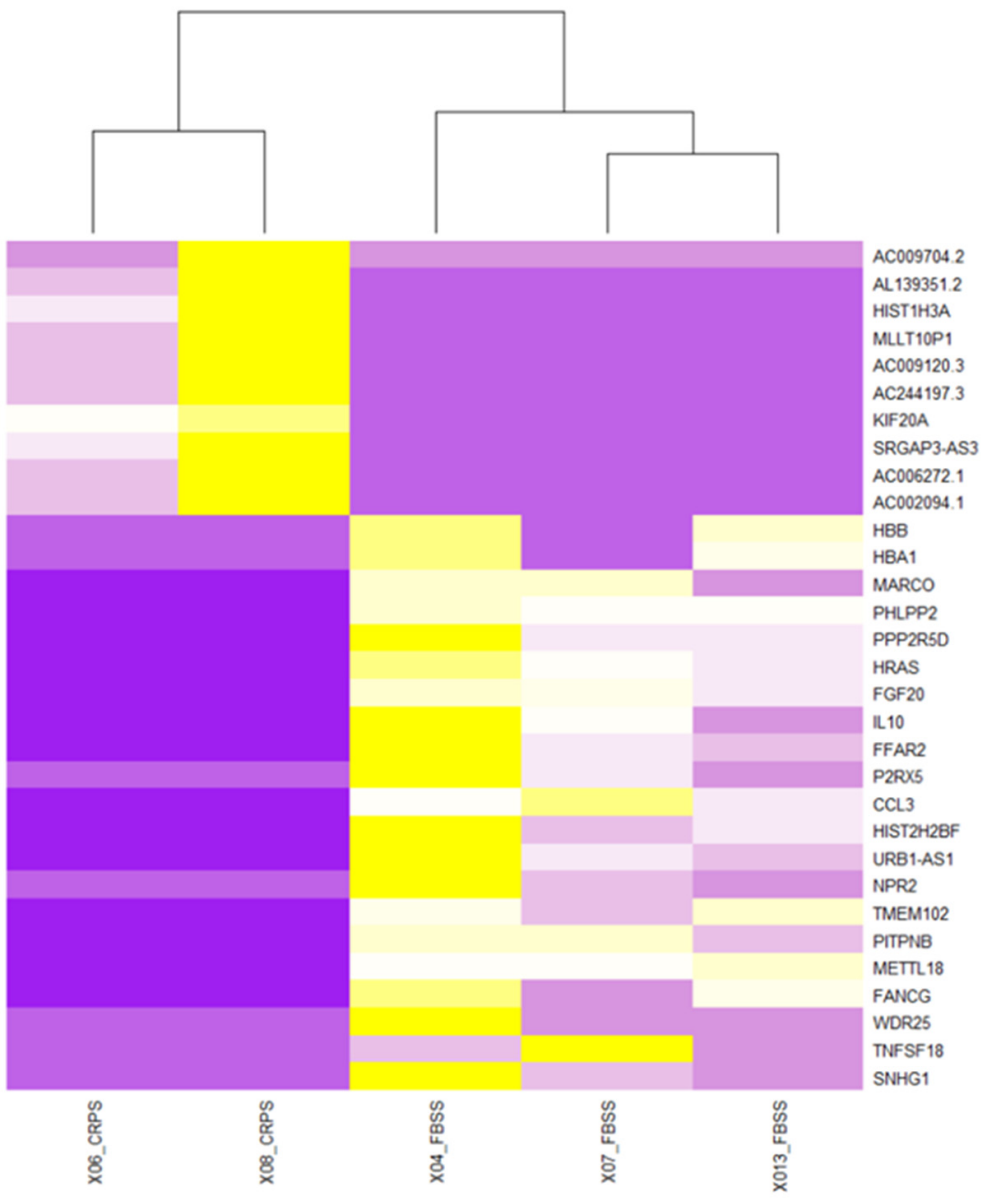
Comparing gene expression in the CSF monocyte population across disease conditions. Plotted here are a selection of genes which were differentially regulated at adj. p < 0.05 according to the DESeq2 algorithm between FBSS and CRPS samples. Colors represent z-scores of TPM values. See Data_File_3 for full gene lists, also for CD4 and CD8 populations. Columns were re-ordered in R using unsupervised clustering with the hclust function (method = ward.D2). Note that CRPS samples are mostly distinguished by lncRNAs (likely noise), while two of the FBSS samples appear to suffer from blood contamination ( *HBB*, *HBA1*).

Bulk sequencing of our sorted populations confirmed that our gating strategy had largely been successful, with each of the populations (CD4+ T cells, CD8+ T cells and monocytes) expressing the expected marker genes ( Figure 3). As reported previously ( Heming *et al.*, 2021), CD4+ T cells expressed high levels of regulatory T cell markers such as FOXP3. Since all three cell types were always processed alongside each other, we would expect the best batch control when comparing across cell types (rather than disease conditions). We therefore first examined which genes were differentially expressed across sorted populations. As expected, monocytes were the most distinct within the bulk RNA-seq dataset we generated, with known monocyte transcripts, like *AIF1* (=IBA1), *TREM2* and *CSF1R*, identified in the top 20 most highly dysregulated genes ( Figure 4). In contrast, any differences we were able to detect across disease conditions (FBSS vs CRPS, Data_File_3) did not appear to rise above technical noise, e.g. with two of the FBSS monocyte samples containing small amounts of blood contamination ( Figure 5). This is unsurprising, given the small sample size, as well as inferior batch control when comparing across individuals: sample processing after each CSF donation proceeded separately until cDNA stage to prevent degradation of small cell numbers at -80C. While samples were once again processed altogether for the remaining library preparation and sequencing, the amount of technical variation introduced by this processing pipeline is a known unknown.

**Figure 6.**
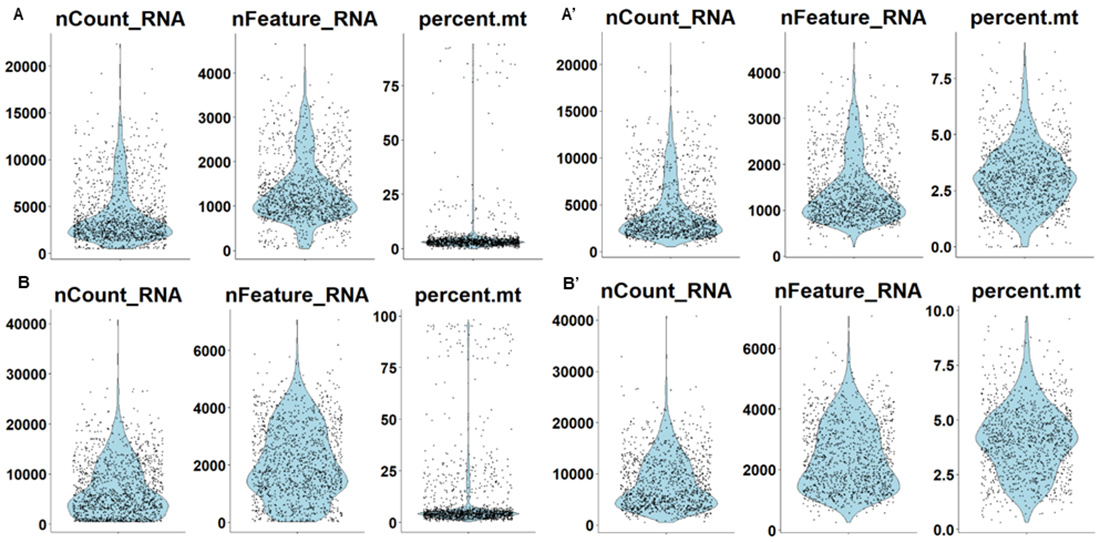
Quality control of CITE-seq data obtained from CSF immune cells from two individuals living with chronic pain. Cells were obtained from a woman with post-herpetic neuralgia ( **A**) and a man with failed back surgery syndrome ( **B**). Data are plotted before ( **A**, **B**) and after filtering ( **A’**, **B’**). Shown are the total number of detected molecules (nCount_RNA), the total number of detected genes (nFeature_RNA) and the percentage of mitochondrial genes within each cell (represented by individual dots). 94% ( **A’**) and 83% ( **B’**) of cells were deemed to pass quality control, containing sufficient molecules and low enough mitochondrial gene counts. The absolute numbers of cells that were taken forward for full analysis were 1222 ( **A’**) and 1149 ( **B’**). Graphs were generated using the R package Seurat (see Supplementary Notebook).

**Figure 7.**
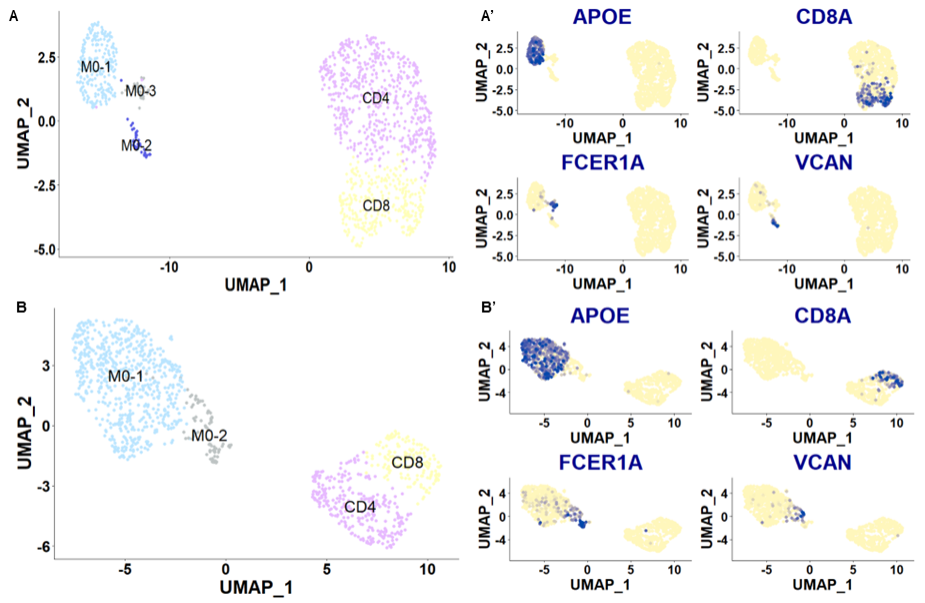
Plots of cell clusters obtained for the two different patient samples. UMAP plots of the CSF cells obtained from the individual with post-herpetic neuralgia ( **A**) and from the individual with failed back surgery syndrome ( **B**). Each dot is a cell. Different cell clusters are indicated by different colors: monocyte populations (M0) in blue & grey, CD4+ T cells in purple and CD8+ T cells in yellow. Clusters were annotated based on marker gene expression shown in **A’** & **B’**. Each dot is a cell. Blue dots indicate cells with positive expression for a given gene.

**Figure 8.**
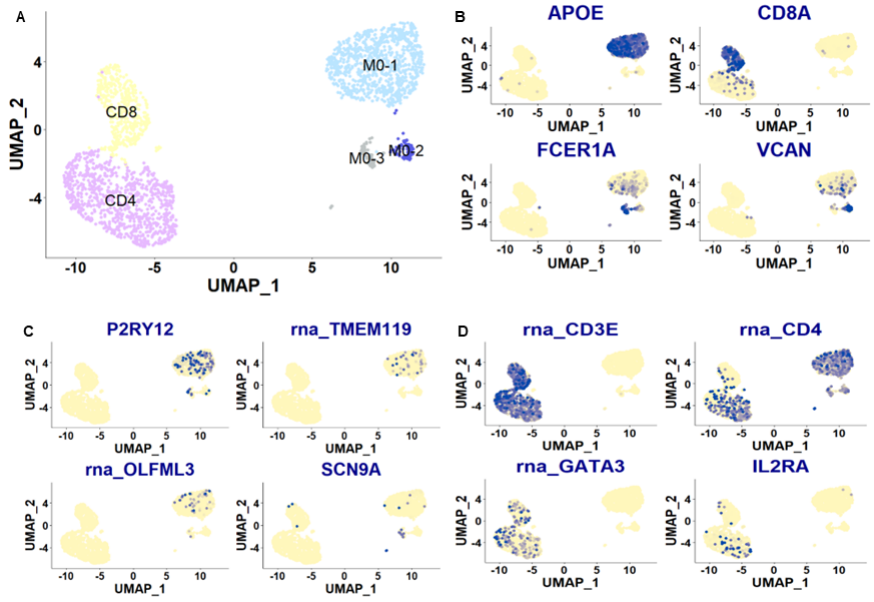
Plots of cell clusters integrated across the two CSF patient samples. UMAP plots of the integrated data. Each dot is a cell. Different cell clusters are indicated by different colors: monocyte (M0) populations in blue & grey, CD4+ T cells in purple and CD8+ T cells in yellow. Clusters were annotated based on marker gene expression shown in **B**. Each dot is a cell. Blue dots indicate cells with positive expression for a given gene. Other relevant marker genes are also plotted in **C** & **D**: genes known for their expression in resident myeloid cells, especially microglia (P2RY12, TMEM119, OLFML3); SCN9A, a voltage-gated sodium channel gene known for its role in nociception; general T cell markers (CD3 and CD4), as well as genes linked to regulatory T cell function (GATA3, IL2RA).

**Figure 9.**
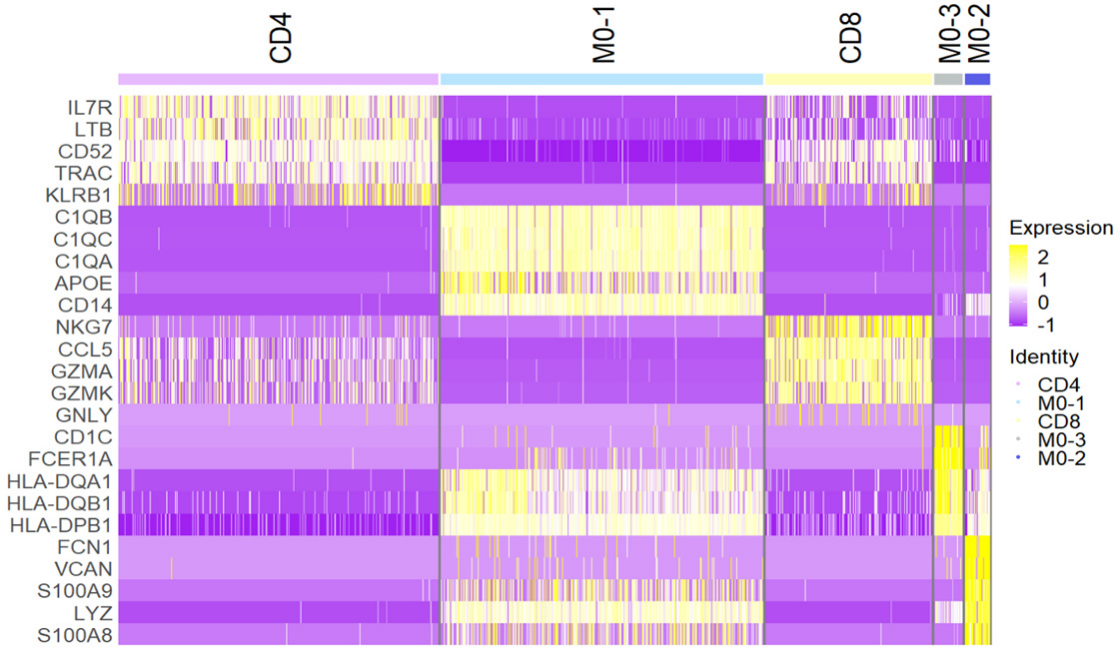
Top five marker genes per cluster. Heatmap of the top five marker genes per cluster. Each line is a cell. The more yellow, the higher the expression of a particular gene in a given cell, the more purple, the lower the expression. Cluster identities are listed at the top: CD4+ T cells in purple (879 cells), Monocyte population 1 (M0-1) in blue (886 cells), CD8+ T cells in yellow (458 cells), Monocyte population 2 in dark blue (69 cells), Monocyte population 3 in grey (79 cells).

**Figure 10.**
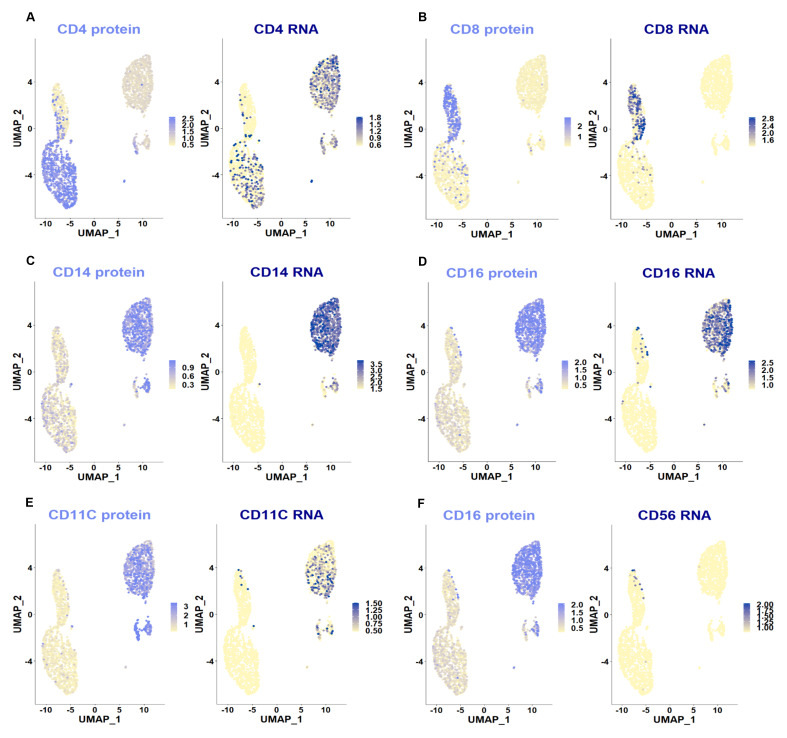
CITE-seq antibody signal juxtaposed with RNA expression data. UMAP plots of the integrated scRNA-seq dataset, split by antibody capture and mRNA signals. As expected, protein expression matched mRNA expression well, with the known exception of CD4, which is widely expressed across immune cell populations at mRNA, but not protein level ( **A**). The sparse CD16 positive cells apparent in the CD8 cluster in **D** are likely CD56 positive T cells: compare **F** for CD16 protein and CD56 mRNA expression. Finally, background antibody binding was high (note the faint CD14 and CD16 signal in T cell populations in **C** & **D**). This is likely due to the need to omit washes in order to preserve cell numbers.

Next, we proceeded to analyze our CITE-seq data derived from one PHN and one FBSS sample. Please see Extended Data for all underlying scripts and Seurat objects. Raw data (including bulk sequencing fastq files) have been deposited on the Gene Expression Omnibus (GEO) under repository: GSE244499. 94% (PHN) and 83% (FBSS) of cells were deemed to pass quality control, containing sufficient molecules and low enough (<10%) mitochondrial gene counts ( Figure 6). 1222 (PHN) and 1149 (FBSS) cells were taken forward to full analysis. The two samples were derived at different times throughout the year and therefore had to be processed separately. Given the lack of batch control, we therefore first analyzed each sample by itself. Unsupervised clustering revealed the expected CD4+ T cell, CD8+ T cell and monocyte populations ( Figure 7). Monocytes spilt into at least two different groups, characterized by expression of *APOE*, *FCER1A* and *VCAN*. The same clusters were apparent when integrating the two datasets using algorithms in Seurat ( Figure 8 & Figure 9). Their mRNA expression correlated well with the antibody tags that were sequenced alongside ( Figure 10). In keeping with our bulk sequencing data and prior literature, expression of myeloid cell markers most commonly associated with microglia ( *P2RY12*, *TMEM119* and *OLFML3*) were once again detectable in our monocyte clusters, primarily within our *APOE*+ monocyte cluster M0-1 ( Figure 8C). Of potential interest to the pain field, transcripts of the sodium channel *SCN9A* appeared detectable in the *FCER1A*+ monocyte cluster M0-3. Mutations in *SNC9A* are known to cause either congenital insensitivity to pain or intense neuropathic pain conditions, like erythromelalgia, depending on where they are located within the gene ( Bennett & Woods, 2014). Finally, as reported before ( Heming *et al.*, 2021), it is clear that genes associated with regulatory T cell function, like *GATA3* and *IL2RA* ( Ohkura *et al.*, 2013), are expressed in CD3+ CSF populations ( Figure 8D).

**Figure 11.**
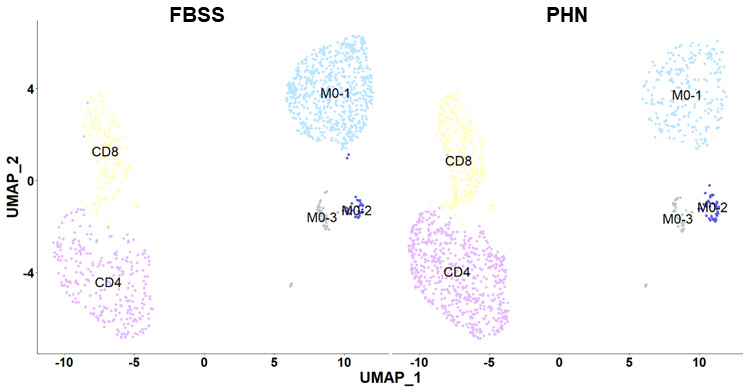
Integrated UMAP plots split by patient ID. UMAP plots of the integrated scRNA-seq dataset, split by patient ID: failed back surgery syndrome (FBSS) and post-herpetic neuralgia (PHN). There were no obvious differences in the identity of the cell populations present across the two conditions. Each dot is a cell. Different cell clusters are indicated by different colors: monocyte (M0) populations in blue & grey, CD4 T cells in purple and CD8 T cells in yellow.

**Figure 12.**
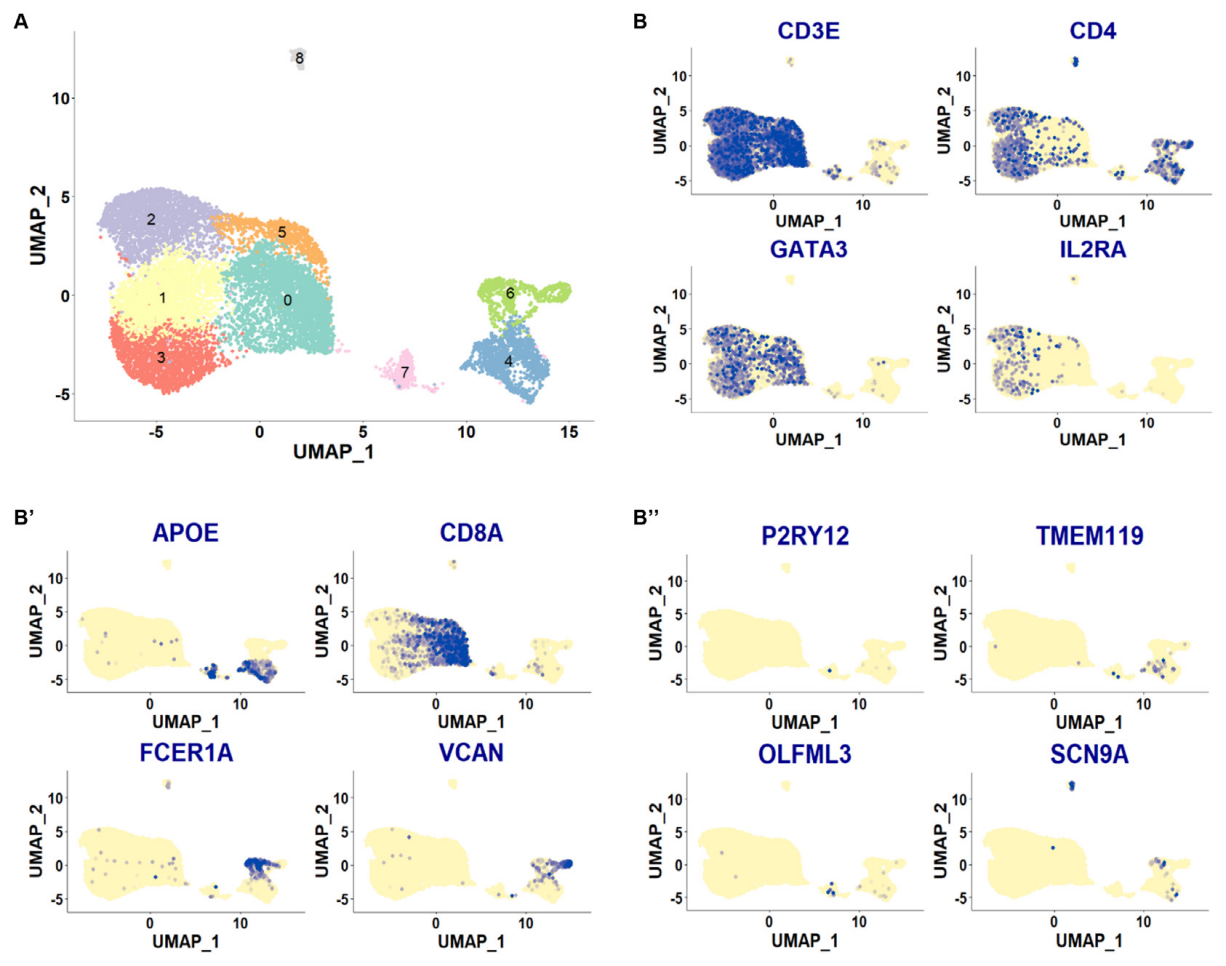
Previously published CSF scRNA-seq data from individuals with idiopathic intracranial hypertension (IIH). UMAP plots of the different cell clusters derived from Heming *et al.* ( Heming *et al.*, 2021). Each dot is a cell. Different cell clusters are indicated by different colors. Marker genes are plotted in B-B’’. Blue dots indicate cells with positive expression for a given gene. Plotted are in B: general T cell markers (CD3 and CD4), as well as genes linked to regulatory T cell function (GATA3, IL2RA); in B’: myeloid cell markers APOE, FCER1A and VCAN as well as T cell marker CD8A; in B’’: genes known for their expression in resident myeloid cells, especially microglia (P2RY12, TMEM119, OLFML3); SCN9A, a voltage-gated sodium channel gene known for its role in nociception.

**Figure 13.**
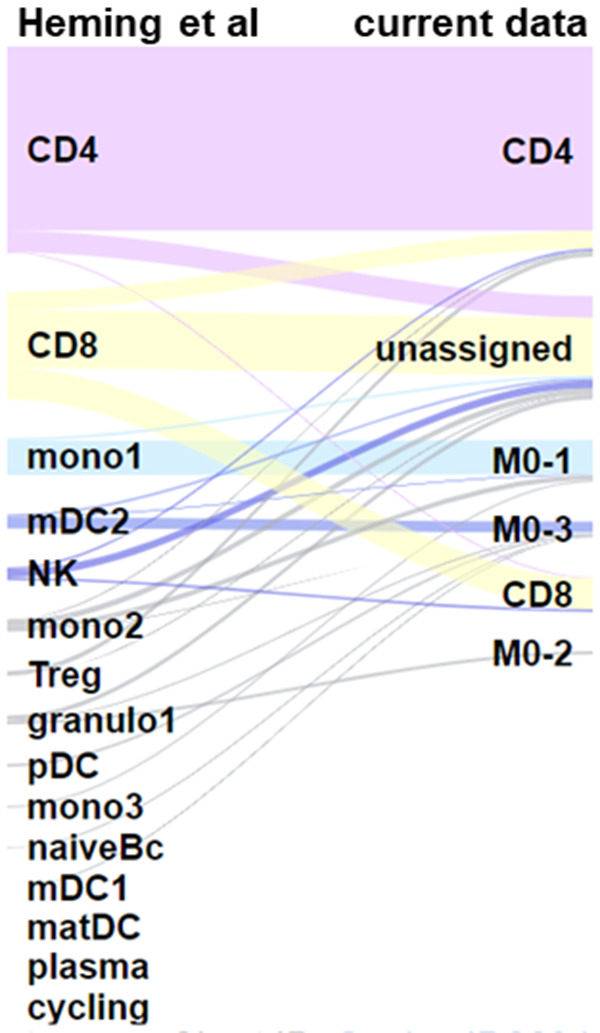
Sankey plot showing how annotation of previously published CSF clusters map onto our scRNA-seq populations. Annotation proposed by Heming *et al.* ( Heming *et al.*, 2021) was compared to our own annotation. The previously published data map well onto our cells, even though the annotation provided by Heming *et al.* contained many more cell types (due to the study including samples in which the blood-brain barrier was disrupted, e.g. as a result of multiple sclerosis).

**Figure 14.**
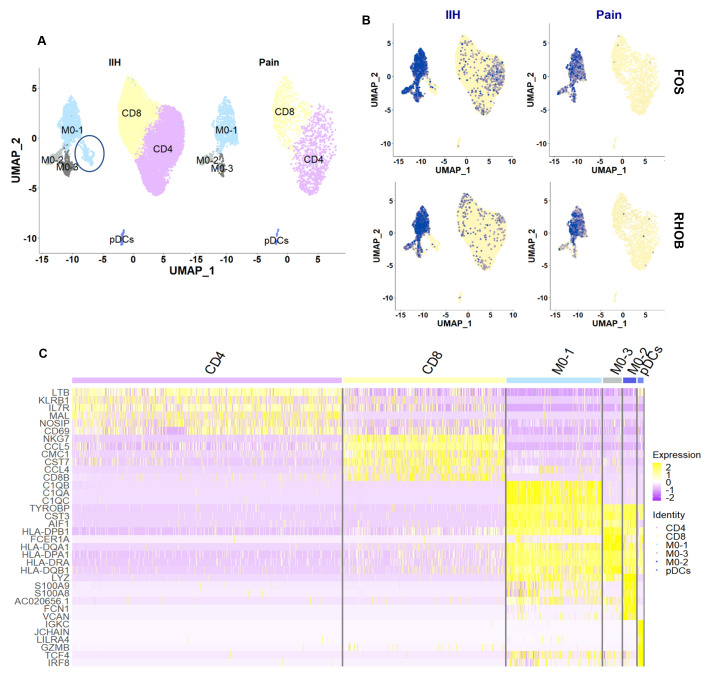
CSF immune cell populations appear similar across neuropathic and non-neuropathic conditions. **A**: UMAP plots of the different cell clusters obtained across our dataset (labelled 'Pain') and a previously published dataset on CSF samples from individuals with IIH ( Heming *et al.*, 2021). Each dot is a cell. Different cell clusters are indicated by different colors: monocyte (M0) populations in light blue & grey, plasmacytoid dendritic cells (pDCs) in dark blue, CD4+ T cells in purple and CD8+ T cells in yellow. Only one cluster appears specific to IIH samples (blue circle). It likely does not consist of a separate cell type, but of M0-1 monocytes that have undergone less cellular stress, as indicated by the absence of immediate early gene expression of FOS and RHOB ( **B**). **C**: Heatmap of the top six marker genes per cluster. Each line is a cell. The more yellow, the higher the expression of a particular gene in a given cell, the more purple, the lower the expression. Cluster identities are listed at the top: CD4 T cells in purple (7224 cells), CD8 T cells in yellow (4339 cells), Monocyte population 1 (M0-1) in blue (2548 cells), Monocyte population 3 (M0-3) in grey (501 cells), Monocyte population 2 (M0-2) in dark blue (335 cells), plasmacytoid dendritic cells (pDCs) in blue (150 cells).

As we found in our bulk sequencing study, there were no clear major differences in cell composition between the two conditions of FBSS and PHN ( Figure 11). Equally, CSF samples derived from individuals with intracranial hypertension (IHH), i.e. individuals without neuropathic pain, looked very similar to our samples when re-analyzed and re-plotted for the same marker genes ( Figure 12). Annotations between the two datasets corresponded well ( Figure 13), with their computational integration suggesting good concordance ( Figure 14). A subset of cells within monocyte population M0-1 appeared less stressed in the IIH dataset, but otherwise all previously identified monocyte (M01-M03) and T cell (CD4 & CD8) populations were present as before, expressing the expected marker genes, including *FCERA1* and *VCAN*.

## Discussion

We used cell sorting and sequencing to analyze the composition of human CSF immune cells derived from seven individuals living with chronic pain that had neuropathic features.

Our results indicate that, as reported previously ( Farhadian *et al.*, 2018; Heming *et al.*, 2021), CSF monocytes produce transcripts traditionally associated with spinal cord microglia. Unfortunately, this means that we cannot be certain whether the proteins that one can assay in CSF derive from local monocytes or whether they were originally produced by microglia within the brain parenchyma. As such, it remains unclear whether analysis of CSF would constitute a useful biomarker for microglial function in humans.

Further, we did not observe any stark differences in immune cell populations between different patient populations: whether people lived with neuropathic pain as a result of failed low back surgery syndrome, postherpetic neuralgia or complex regional pain syndrome – their CSF immune cells, at least at a superficial level, were indistinguishable from each other and from CSF immune cells found in people with idiopathic intracranial hypertension, i.e. people that were presumably neuropathic pain free. This is not particularly unexpected – since the blood brain barrier remains intact in all these conditions, there is no reason to suppose that CSF immune cells would differ markedly, even from those found in healthy individuals. And if they were to differ, one would expect the alterations to be of more moderate or even small effect sizes, i.e. necessitate sample sizes of at least n = 50 per condition.

Despite the small-scale nature of our study, we hope that it may prove useful to the scientific community through aggregation with other existing and future datasets. It is not straightforward to set up an experimental pipeline that allows for analysis of CSF immune cells within a few hours of donation. And yet, this is the time-frame required to avoid significant monocyte loss ( de Graaf *et al.*, 2011). It would be a shame if any such data were lost in file-drawers, particularly when they include transcriptional information which can be relatively well-integrated across studies, at least at the level of cell type. Indeed, we have demonstrated as part of this work that our data mapped concordantly onto CSF scRNA-seq results published by one of the leading groups in human CSF transcriptional analysis ( Heming *et al.*, 2021; Heming *et al.*, 2019).

Finally, this study has encouraged us to delve deeper into what we already know and what we still stand to learn about microglia in neuropathic pain states. We feel that more data in humans are desperately needed, not least since microglial dysregulation is at its most obvious in animal models that are traumatic in nature, i.e. involve directly cutting into peripheral nerves. And while such injuries can occur in humans, the majority of human neuropathic pain does not result from surgical trauma ( Finnerup *et al.*, 2021), but is instead caused by conditions like diabetes, sterile nerve entrapment or shingles, as in the case of postherpetic neuralgia, analyzed here via CITE-seq. Human data can be acquired post-mortem, via imaging or using CSF. Regarding post-mortem analysis, scRNA-seq of human spinal cord is making great strides ( Yadav *et al.*, 2023), but a link to pathology and especially pain phenotyping remains understandably more elusive. Meanwhile, our results clearly demonstrate that using CSF will be complicated: even if some of the protein content in the fluid reflects underlying microglial pathology, it is likely to be a small signal, dwarfed by changes in CSF monocyte number or function. Whether monocytes themselves are dysregulated in neuropathic pain states remains to be explored in suitably powered studies. In the meantime, we speculate that the fastest, most efficient way to fully resolve the question of whether microglia are activated in human neuropathic pain states might be via the use of novel PET imaging markers. Not least since it would not involve any invasive procedures. We keenly anticipate the development of these technologies and look forward to such data becoming available.

## Data Availability

We used a previously published dataset for comparison ( Heming *et al.*, 2021), which is available on GEO under accession GSE163005. Sequencing data generated as part of this manuscript have been deposited on GEO (accession number GSE244499). All other data are provided as extended data files on the Open Science Framework: https://doi.org/10.17605/OSF.IO/F4ZRQ They are available under the terms of the Creative Commons Zero "No rights reserved" data waiver (CC0 1.0 Public domain dedication). The following files and folders are contained within the repository: FACS_data. Flow cytometry data for all seven CSF samples analysed as part of this study. This includes .fcs files, as well as pdfs of the gates originally used when sorting the cells. processed_data_bulkRNAseq Data_File_1.xlsx. Bulk-sequencing alignment statistics. Data_File_2.xlsx. Processed bulk sequencing data (TPM values and limma output) Data_File_3.xlsx. Processed bulk sequencing data (DEseq2 output) processed_data_CITEseq Supplementary_Notebook.Rmd. R script used to analyse CITE-seq data and generate Figures 6–14. Supplementary_Notebook_html. The html output of the Supplementary_Notebook, which shows the results of each line of script. FBSS_umap_011.Robj. Seurat Object containing FBSS sample data. PHN_umap_005.Robj. Seurat Object containing FBSS sample data. integrated_umap.Robj. Seurat Object containing the two samples integrated. Heming&Zoe_integrated.Robj. Seurat Object containing our two samples integrated with IIH samples derived from Heming *et al.*
