# Supplementary material for: Cerebrospinal fluid immune cells appear similar across neuropathic and non-neuropathic pain conditions
Source: Wellcome Open Res. 2023 Oct 25;8:493. [Version 1] doi: 10.12688/wellcomeopenres.20153.1 (PMC11069048; doi:10.12688/wellcomeopenres.20153.1)
Supplement: Peer review history

# Reviewer report: Reviewer response for version 1

**DOI:** 10.21956/wellcomeopenres.22315.r71914

**Published:** 2024-05-03

**Authors:** Margarita Calvo

**Affiliations:** 1 Department of Physiology, Faculty of Biological Sciences, Pontificia Universidad Católica de Chile, Santiago, Chile

Thank you for offering the opportunity to review this very interesting paper. The idea to examine the cell population of the CSF of neuropathic pain patients is very clever and could lead to a potential biomarker.

 The authors examined the CSF of patients with failed back surgery syndrome (FBSS), complex regional pain syndrome (CRPS) and postherpetic neuralgia (PHN). They utilized FACS analysis and bulk sequencing to compare major immune cell populations across FBSS or CRPS and CITE sequencing to compare a sample from FBSS or PHN, as well as with a previously published scRNAseq CSF dataset from idiopathic intracranial hypertension (IIH) patients.

 The authors did not find a clear major difference in cell composition among the conditions. However, as they acknowledge, the sample was small and not power to detect subtle differences. Having said this, this dataset is still of interest for the pain community and will surely add on previous and future research.

 The introduction is very clear and updated. The methods and results are well and completely presented. The strengths and limitations of the study are well discussed.

 I would only suggest the authors to add on the DN4 data from the patients. Do the DN4 scoring correlates with the cell signature? The same for the VAS, does the intensity of pain have any effect on the immune cell signature?

 I hope this study will be an incentive for this and other groups to further investigate immune cell signature in different pain and painless conditions.

Is the work clearly and accurately presented and does it cite the current literature?

Yes

If applicable, is the statistical analysis and its interpretation appropriate?

Yes

Are all the source data underlying the results available to ensure full reproducibility?

Yes

Is the study design appropriate and is the work technically sound?

Yes

Are the conclusions drawn adequately supported by the results?

Yes

Are sufficient details of methods and analysis provided to allow replication by others?

Yes

Reviewer Expertise:

Neurobiology of pain

I confirm that I have read this submission and believe that I have an appropriate level of expertise to confirm that it is of an acceptable scientific standard.

# Reviewer report: Reviewer response for version 1

**DOI:** 10.21956/wellcomeopenres.22315.r73201

**Published:** 2024-02-20

**Authors:** Nathan Fiore

**Affiliations:** 1 Department of Physiology, Translational Neuroscience Facility, School of Biomedical Sciences, University of New South Wales, Sydney, Australia

The article presented from Hore, Z., Denk, F. and colleagues investigated immune cell populations within CSF of patients experiencing failed back surgery syndrome (FBSS), complex regional pain syndrome (CRPS) and postherpetic neuralgia (PHN). The authors utilized FACS analysis and bulk sequencing to compare major immune cell populations across FBSS or CRPS and CITE sequencing to compare a sample from FBSS or PHN, as well as with a previously published scRNAseq CSF dataset from idiopathic intracranial hypertension (IIH) patients.

 The authors were able to characterize CD4, CD8 T cells and monocyte populations and their signatures within the CSF, but found no clear major differences in cell composition between any of the various conditions. 

 Overall, this article gives sufficient background in the introduction, methods are detailed and results are clear and well presented. The authors clearly acknowledge the strengths and limitations of the study, and I agree that this dataset will be of interest to the pain community.

 I have two minor points I'd like the authors to address;

 Sex differences in chronic pain is always of particular interest in the pain community. The authors were unable to detect any differences across FBSS vs CRPS within the bulk RNA-seq dataset. Given CITE-seq was run on CSF sample from a woman with PHN and a man with FBSS, could the authors include a male vs female comparison from the combined FBSS and CRPS dataset? I understand with the small sample size and variances in pain conditions it would be unlikely to detect any differences, though I think it would be of interest to readers to confirm whether there are any differences or not.

 I am curious whether this monocyte population expressing microglial markers is observed in healthy populations? I understand that this IIH patient data set is utilized as a non-neuropathic pain cohort (or non-inflammation cohort in Heming et al., 2021), though I am unsure whether this immune CSF would be reflective of healthy controls. Does the HIV- cohort utilized in Farhadian et al., 2018 have a similar monocyte signature to these cohorts?

Is the work clearly and accurately presented and does it cite the current literature?

Yes

If applicable, is the statistical analysis and its interpretation appropriate?

Yes

Are all the source data underlying the results available to ensure full reproducibility?

Yes

Is the study design appropriate and is the work technically sound?

Yes

Are the conclusions drawn adequately supported by the results?

Yes

Are sufficient details of methods and analysis provided to allow replication by others?

Yes

Reviewer Expertise:

Neuropathic pain, neuroimmunology

I confirm that I have read this submission and believe that I have an appropriate level of expertise to confirm that it is of an acceptable scientific standard.

# Response

**Published:** 2024-02-20

**Authors:** Franziska Denk

**Affiliations:** King's College London (Subscriptions), UK

Thank you so much for taking the time to review our manuscript and for your interesting comments. Myself and the other authors really appreciate it.  The simple answer first: yes, Farhadian et al. see the same monocyte population (containing transcripts best described in microglia) in their HIV+ patients. Completely healthy CSF is hard to come by, so I'm less sure about that. However, currently, there is no evidence to indicate that these monocytes are in any way pathological.  Regarding sex differences - a very good question, of course. However, really difficult to answer with just n = 1. In my experience looking at sequencing data, only a few X and Y linked genes are always very prominently different between male and female samples. As soon as you ask questions about other genes, you will need quite significant samples sizes to detect any differences. As an example, here ( https://osf.io/rmcve/files/osfstorage/65d465f3c3ab4906ef46b7ff) I have used our data to plot for you the expression of X-&Y-linked genes (which clearly segregate), as well as the expression of some genes found on the X chromsome with known roles in female-predominant immune-mediated diseases. The latter already no longer segregate by sex. Generally, I fear that we all have a tendency to underestimate the number of samples needed to reliably detect sex differences, even for the most simple questions. For example, women, on average, weigh less than men. But that is a moderate effect (as defined by Cohen), and you'd actually need to weigh ~50 women and 50 men to be powered to detect this difference reliably in your samples. It's sobering, especially when we think of the kind of complex questions we seek to answer (e.g. about differential pain perception and/or processing between the genders).
